# Multimodal synergisms in host stimuli drive landing response in malaria mosquitoes

**DOI:** 10.1038/s41598-021-86772-4

**Published:** 2021-04-01

**Authors:** Manuela Carnaghi, Steven R. Belmain, Richard J. Hopkins, Frances M. Hawkes

**Affiliations:** Department of Agriculture Health and Environment, Natural Resources Institute, University of Greenwich at Medway, Kent, ME7 4TB UK

**Keywords:** Animal behaviour, Entomology, Malaria

## Abstract

*Anopheles* mosquitoes transmit malaria, which affects one-fifth of the world population. A comprehensive understanding of mosquito behaviour is essential for the development of novel tools for vector control and surveillance. Despite abundant research on mosquito behaviour, little is known on the stimuli that drive malaria vectors during the landing phase of host-seeking. Using behavioural assays with a multimodal step approach we quantified both the individual and the combined effect of three host-associated stimuli in eliciting landing in *Anopheles coluzzii* females. We demonstrated that visual, olfactory and thermal sensory stimuli interact synergistically to increase the landing response. Furthermore, if considering only the final outcome (i.e. landing response), our insect model can bypass the absence of either a thermal or a visual stimulus, provided that at least one of these is presented simultaneously with the olfactory stimuli, suggesting that landing is the result of a flexible but accurate stimuli integration. These results have important implications for the development of mosquito control and surveillance tools.

## Introduction

Mosquitoes can transmit a range of pathogens that collectively cause the death of over 700,000 people each year^1^. Of these, malaria, which is transmitted by mosquitoes of the *Anopheles* genus, causes the highest annual mortality^1^. Insecticidal bed nets and indoor spraying of insecticides have significantly reduced malaria in endemic regions by targeting the mosquito vector; these two interventions account for nearly 80% of the estimated 663 million cases averted between 2000 and 2015^2^. However, there has been a worrying increase in the strength and distribution of physiological and behavioural resistance to insecticides within vector populations, compromising the long-term utility of these control measures^3–5^. Thus, there is an urgent need for new vector control and surveillance tools, the most effective of which must be designed taking into account the behaviour and ecology of the vector^6,7^.

Although vector behaviour has been intensively studied, research has often focussed on responses to host odour^8,9^, which is only one of the many host cues that shape host-seeking behaviour^10^. Initial activation and long-range orientation (over many metres) is mediated by volatile odours and carbon dioxide^10^, and the detection and behavioural responses to these stimuli are well-characterised in several disease vector species^8,11–13^. Within the range of visual resolution, which depends on the species and the size of the object, but can be up to 5 m away, visual stimuli are also attractive^14^. In addition, visual feedback is crucial for mosquito orientation and upwind flight as they use the optomotor anemotaxis mechanism to adjust flight manoeuvres and their orientation^14^. When in a plume of host odour, *Anopheles coluzzii* rapidly decrease their mean ground speed and closely approach visually conspicuous targets to within a few centimetres, but do not make contact^15^. At the very close range, thermal and humidity gradients detected by thermoreceptors on the mosquito antennae^16,17^, proboscis^18^, and maxillary palps^19^, and hygroreceptors on the antennae^20,21^, influence the final stages of close-range orientation and landing^9,10,22,23^. Recent studies have characterised the function of heat receptors in both *Aedes aegypti* and *An. gambiae *sensu stricto mosquitoes, elucidating their function in heat avoidance and heat-seeking, respectively^24,25^. However, the response to thermal stimuli varies when there is simultaneous stimulation of olfactory receptors. In the presence of human odour, *An. gambiae s.s.* mosquitoes fly faster, with more convoluted flight trajectories and land significantly more often on a surface if it is heated to 34 °C, representative of human skin temperature^26^.

Landing, the final stage of host-seeking behaviour, is the crucial moment of vector-host contact. However, research to systematically unravel the effect of different sensory modalities on this behaviour has been relatively neglected^9,27,28^. Published studies present only the response to one or two stimuli at a time^15,26,29,30^, or focus on diurnal vectors of the genus *Aedes*, which display some different behaviours to the nocturnal *Anopheles*^31,32^. Thus, in this study we set out to determine the relative importance of thermal, visual and odour cues in mediating landing behaviour of the primary vector of malaria in sub-Saharan Africa, *Anopheles coluzzii*. We used a multimodal step approach, where stimuli were presented first alone, then in combination to facilitate a quantification of the effect of each stimulus and how the behavioural effect changed as a result of multimodal integration. We report here the strong synergistic effects between thermal and odour cues, and among all three host-associated cues, which amplified the landing response, and the crucial role of host odour in enabling landing behaviour. Understanding mosquito responses to specific host cues provides insights into behaviours that could be exploited to improve current surveillance and control tools, or provide the basis for new behaviourally-based interventions.

## Results

**Figure 1:**
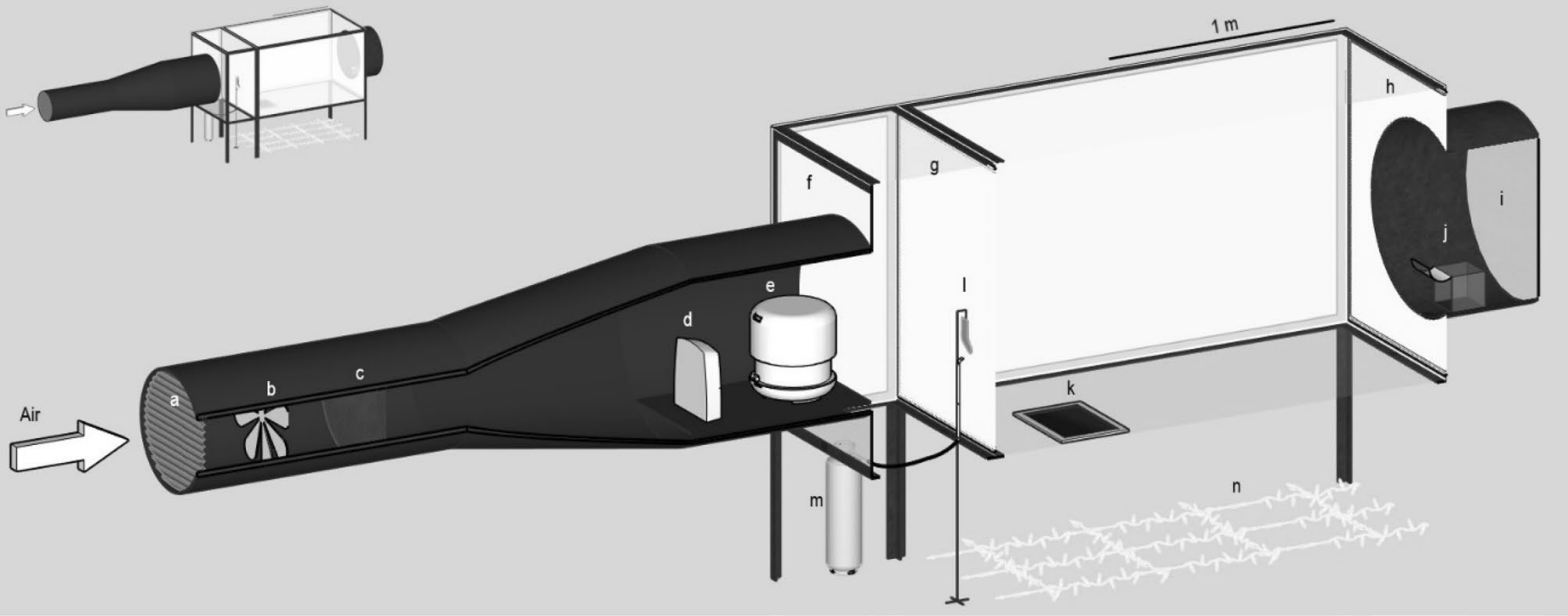
Wind tunnel and flight arena schematic. (a) shutter, (b) impelling fan, (c) charcoal filters, (d) fan heater, (e) atomising humidifier, (f) brushed cotton screen, (g) upwind white net screen, (h) downwind white net screen, (i) terminal downwind netting, (j) release cage, (k) landing target, (l) odour delivery platform, with worn sock and carbon dioxide release tube, (m) carbon dioxide tank, (n) lighting array.

The study was carried out in a large wind tunnel (Fig. 1)^15^, where mosquitoes were released at the downwind end and a target was presented at the upwind end. Twelve different treatments were tested, representing all possible combinations of the following factors: visual variables (the target was either transparent or visually conspicuous), odour variables (presence or absence of volatile odours from a human foot plus constant carbon dioxide) and thermal variables (the target was set at either 25 °C, 35 °C, or 45 °C). The number of mosquitoes recovered from the surface of the target and in different parts of the wind tunnel flight arena were counted. A total of 124 assays were conducted, a minimum of ten replicates were carried out for each treatment, releasing a mean number of 25 ± 1 (SEM) mosquitoes per replicate, thus using a total of 3074 mosquitoes.

**Figure 2:**
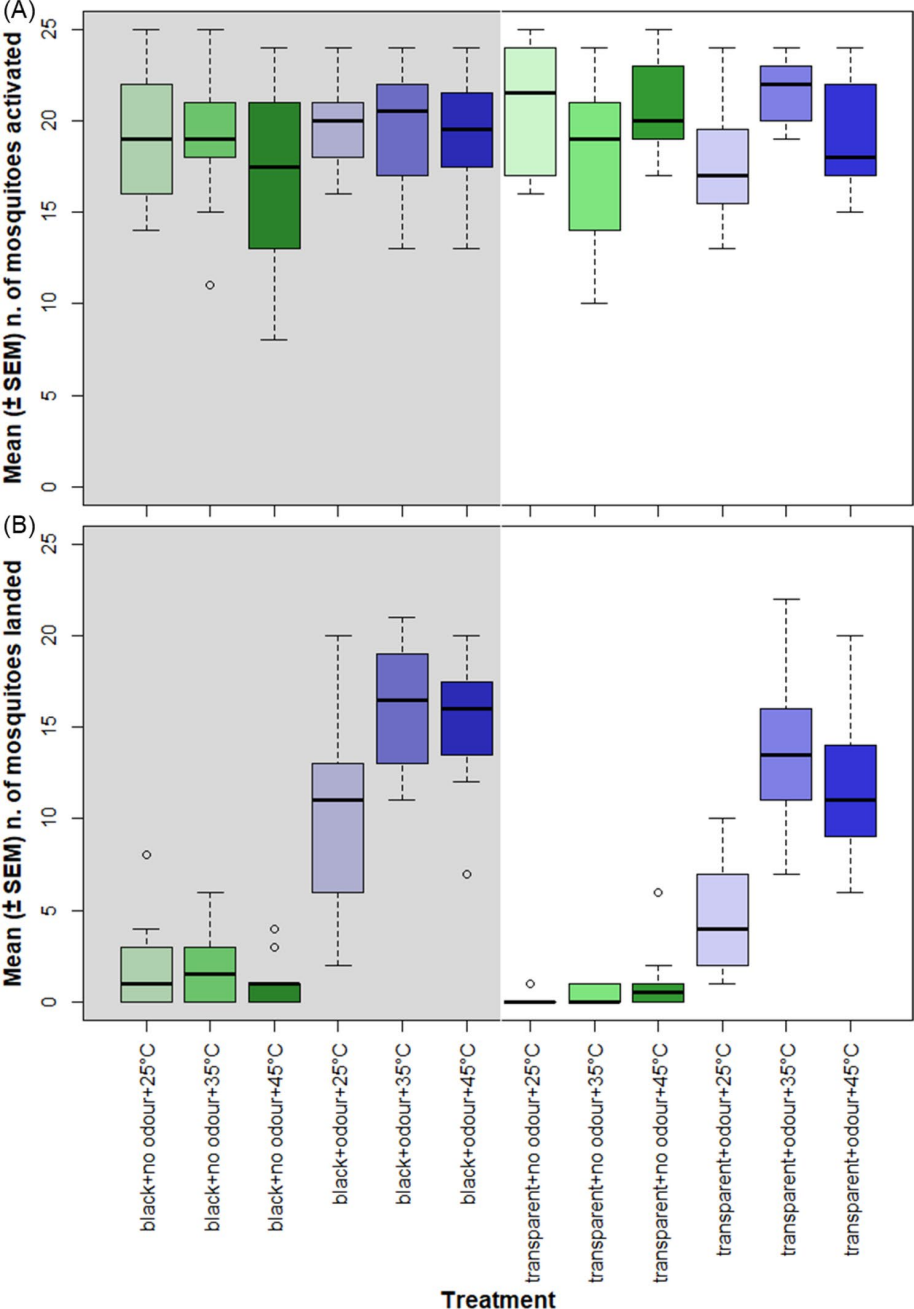
Mean number (± SEM) of mosquitoes activated (**A**) and mean number (± SEM) of mosquitoes found on the landing surface (**B**) for each treatment. Bold black bars indicate the median; upper and lower limits of a box indicate the interquartile range; whiskers indicate the maximum and minimum data points, excluding outliers, which are indicated by the circles. The y-axis shows the twelve different treatments, where “black” and “transparent” indicate the different visual cues offered, “no odour” and “odour” indicate the absence or presence of host odour, and the temperature indicates the thermal cue used. Blue bars indicate host odour was present during assays, green bars indicate it was absent. Paler colours denote lower temperatures and darker colours indicate higher temperatures. Background colour indicates the presence (grey) or absence (white) of the visual cue.

The presence or absence of the odour cue had no significant effect on the activation rate (number of mosquitoes which left the release cage during an assay) (χ^2^ = 1.00, d.f. = 1, *P* = 0.53). Similarly, no effect was found on the activation rate independently of the visibility of the landing target (χ^2^ = 1.46, d.f. = 1, *P* = 0.45) or the temperature of the target (χ^2^ = 0.93, d.f. = 2, *P* = 0.83). Thus, none of the host-associated stimuli presented had a significant effect on the activation behaviour of *An. coluzzii* (Fig. 2A).

### Landing overview

Overall, mosquitoes were equally recovered in all quadrants of the landing target and no significant difference was found between landings on the downwind quadrants compared to the upwind quadrants (χ^2^ = 0.86, *P* = 0.64). This allowed verification that the flight arena conditions were symmetrical.

When considered as independent factors, all three variables tested had a significant effect on driving the landing response of *An. coluzzii* (analysis of deviance, for temperature: χ^2^ = 40.92, d.f. = 2, *P* < 0.001, for visibility: χ^2^ = 26.25, d.f. = 1, *P* < 0.001, for host odour: χ^2^ = 503.97, d.f. = 1, *P* < 0.001). Presence or absence of host odour had the strongest effect on landing behaviour which was enhanced by the addition of visual and thermal cues. Overall, the highest number of catches was obtained in assays where all the three cues were presented together (Fig. 2B).

It is interesting to note that independent of other variables, no difference was ever observed when comparing the number of landings between corresponding treatments where the surfaces were at 35 °C and 45 °C. This indicated that as long as the surface was warmer than the environment (at 25 °C), it elicited a similar landing response, and even the highest temperature did not produce a behavioural cut-off in the landing response.

To establish the role of each host-associated cue and its relative importance in driving landing behaviour, we undertook a series of comparisons between each permutation of possible treatment combinations (Table 1).

**Table 1:** Tukey contrast comparisons between mean landing rates for each permutation of possible treatment combinations.

Factor compared	Treatment 1	Mean (± SEM)	Treatment 2	Mean (± SEM)	z-value	*P* value
Odour cue	**O** + T + 25 °C	4.8 (± 0.88)	**N** + T + 25 °C	0.2 (± 0.13)	4.37	*P* < 0.001***
Visual cue	N + **B** + 25 °C	2.0 (± 0.82)	N + **T** + 25 °C	0.2 (± 0.13)	− 3.08	*P* = 0.07
Thermal cue	N + T + **25 °C**	0.2 (± 0.13)	N + T + **35 °C**	0.4 (± 0.16)	0.80	*P* = 1
Thermal cue	N + T + **25 °C**	0.2 (± 0.13)	N + T + **45 °C**	1.1 (± 0.59)	2.20	*P* = 0.49
Thermal cue	N + T + **35 °C**	0.4 (± 0.16)	N + T + **45 °C**	1.1 (± 0.59)	1.71	*P* = 0.83
Odour cue in presence of thermal cue	**O** + T + 35 °C	13.3 (± 1.41)	**N** + T + 35 °C	0.4 (± 0.16)	6.79	*P* < 0.001***
Odour cue in presence of thermal cue	**O** + T + 45 °C	11.8 (± 1.24)	**N** + T + 45 °C	1.1 (± 0.59)	7.21	*P* < 0.001***
Odour cue in presence of visual cue	**O** + B + 25 °C	10.4 (± 1.77)	**N** + B + 25 °C	2.0 (± 0.82)	6.30	*P* < 0.001***
Odour cue in presence of visual and thermal cue	**O** + B + 35 °C	15.9 (± 1.05)	**N** + B + 35 °C	1.7 (± 0.62)	8.21	*P* < 0.001***
Odour cue in presence of visual and thermal cue	**O** + B + 45 °C	15.2 (± 1.00)	**N** + B + 45 °C	1.2 (± 0.42)	8.14	*P* < 0.001***
Visual cue in presence of odour cue	O + **B** + 25 °C	10.4 (± 1.77)	O + **T** + 25 °C	4.8 (± 0.88)	− 4.17	*P* < 0.001***
Visual cue in presence of thermal cue	N + **B** + 35 °C	1.7 (± 0.62)	N + **T** + 35 °C	0.4 (± 0.16)	− 2.57	*P* = 0.25
Visual cue in presence of thermal cue	N + **B** + 45 °C	1.2 (± 0.42)	N + **T** + 45 °C	1.1 (± 0.59)	− 0.20	*P* = 1
Visual cue in presence of thermal cue and odour cue	O + **B** + 35 °C	15.9 (± 1.05)	O + **T** + 35 °C	13.3 (± 1.41)	− 1.18	*P* = 0.99
Visual cue in presence of thermal cue and odour cue	O + **B** + 45 °C	15.2 (± 1.00)	O + **T** + 45 °C	11.8 (± 1.24)	− 1.69	*P* = 0.84
Thermal cue in presence of visual cue	N + B + **25 °C**	2.0 (± 0.82)	N + B + **35 °C**	1.7 (± 0.62)	− 0.47	*P* = 1
Thermal cue in presence of visual cue	N + B + **25 °C**	2.0 (± 0.82)	N + B + **45 °C**	1.2 (± 0.42)	− 1.35	*P* = 0.96
Thermal cue in presence of visual cue	N + B + **35 °C**	1.7 (± 0.62)	N + B + **45 °C**	1.2 (± 0.42)	− 0.90	*P* = 1
Thermal cue in presence of odour cue	O + T + **25 °C**	4.8 (± 0.88)	O + T + **35 °C**	13.3 (± 1.41)	5.64	*P* = 0.001***
Thermal cue in presence of odour cue	O + T + **25 °C**	4.8 (± 0.88)	O + T + **45 °C**	11.8 (± 1.24)	4.92	*P* = 0.001***
Thermal cue in presence of odour cue	O + T + **35 °C**	13.3 (± 1.41)	O + T + **45 °C**	11.8 (± 1.24)	− 0.76	*P* = 1
Thermal cue in presence of visual cue and odour cue	O + B + **25 °C**	10.4 (± 1.77)	O + B + **35 °C**	15.9 (± 1.05)	2.69	*P* = 0.19
Thermal cue in presence of visual cue and odour cue	O + B + **25 °C**	10.4 (± 1.77)	O + B + **45 °C**	15.2 (± 1.00)	2.47	*P* = 0.30
Thermal cue in presence of visual cue and odour cue	O + B + **35 °C**	15.9 (± 1.05)	O + B + **45 °C**	15.2 (± 1.00)	− 0.33	*P* = 1

*O* Odour, *N* No odour, *T* transparent, *B* Black.

In bold is highlighted the changing variable in each comparison.

***Denotes significant difference between the treatments.

**Figure 3:**
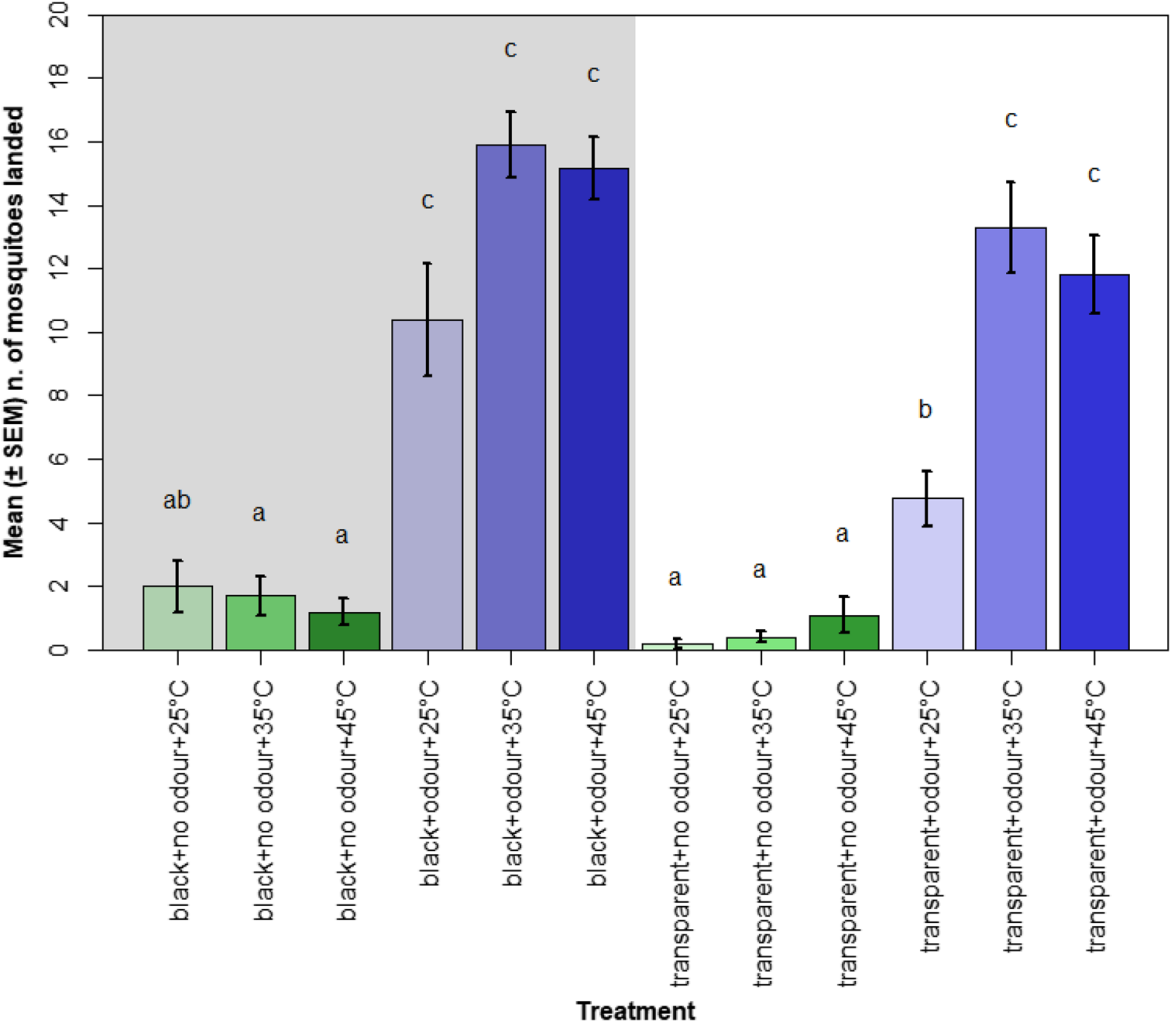
Mean number of mosquitoes (± SEM) found on the landing surface in each treatment. The y-axis shows the twelve different treatments, where “black” and “transparent” indicate the different visual cues offered, “no odour” and “odour” indicate the absence or presence of host odour, and the temperature indicates the thermal cue used. Blue bars indicate host odour was present during assays, green bars indicate it was absent. Paler colours denote lower temperatures and darker colours indicate higher temperatures. Background colour indicates the presence (grey) or absence (white) of the visual cue. Different letters denote significant differences between treatments (Tukey contrast comparisons test, *P* < 0.01).

Significantly more mosquitoes landed on the target when host odour was the only cue presented compared with the number of landings when the cue was missing. On the other hand, the visibility of the target, when presented alone, did not significantly affect the number landings on the target. Although a small number of individuals landed on the black target, this was not significantly different from the mean number of mosquitoes that landed on a transparent target. Similarly, when the temperature of the target was the only cue offered, it did not influence landing behaviour relative to a transparent target at 25 °C. Collectively, these results show that when no other stimuli are present, neither a visual nor a thermal stimulus alone are sufficient to induce landing (Fig. 3).

### Interactions between host odour and other host-associated cues

The addition of host odour to either the thermal cue or to the visual cue had a significant effect on the landing behaviour, as it greatly increased the number of mosquitoes recovered on the target in the respective treatments. Furthermore, significantly more mosquitoes landed on the target when all three cues were presented together when compared to treatments where the odour cue was absent, or when compared to the treatment where only the odour cue was presented. However, the number of mosquitoes caught on the surface when all three cues were offered was not significantly higher than the number caught when either the visual or thermal cue were missing. Thus, odour always plays a major role in triggering the behaviours that take mosquitoes to land and its absence results in low landing proportions. Visual and thermal cues increase landings if introduced as the only other cue presented along with host odour, while their role becomes secondary when host odour and either one of the visual or thermal cue are already present, increasing the landing behaviour in a minor manner.

### Interaction between visual cue and other host-associated cues

More mosquitoes landed on black target surfaces in the presence of host odour compared to the number that landed on transparent targets in the presence of host odour. However, no effect was recorded on the landing behaviour when, in the absence of host odour, the mosquitoes were presented with a black surface at 35 °C or 45 °C compared with landings respectively on a transparent surface at 35 °C or 45 °C. This indicates that, in absence of host odour, the visibility of a heated target surface does not influence the landing behaviour. Equally, in the presence of host odour, no significant difference was found in the number of landings between treatments where the visually conspicuous target surface was set at 35 °C or 45 °C when compared with a treatment which was identical apart from having a transparent surface. These results indicate that the visibility of the target did not increase the landing behaviour if the other two factors, i.e. host odour and thermal cue, were present. Thus, mosquitoes can bypass the absence of the visual cue if sufficient non-visual host-associated stimuli are present.

### Interaction between thermal cue and other host-associated cues

In treatments where host odour was absent and the target surface was visually conspicuous, the temperature of the surface did not influence the landing behaviour. Specifically, no significant effect was found when comparing the number of mosquitoes caught on a black surface at 25 °C with the number caught on the same surface at 35 °C and 45 °C. On the other hand, when the thermal cue was provided simultaneously with host odour, a substantial effect on the landing response was recorded. A higher number of mosquitoes were caught in the treatment where the transparent target was set at 35 °C and 45 °C and was presented with host odour when compared to the same target at 25 °C. Furthermore, the number of landings were not different in treatments where the black surface was set at either 35 °C or 45 °C and was presented with host odour compared to the respective treatment where the temperature was 25 °C. These results indicate that a thermal cue does not increase landing if other host-associated stimuli, i.e. host odour and visual cue, are present. Thus, mosquitoes can compensate for the absence of the thermal cue if they receive other sufficient information to locate the host.

### Quantification of the role of each stimulus on triggering landing behaviour and synergistic effect of combined stimuli

As human body temperature was the most behaviourally relevant thermal cue, and no significant difference was found between the number of mosquitoes recovered on target surfaces set at 35 °C and at 45 °C, the latter temperature was removed for this analysis. We calculated what proportion of landing response was attributable to each stimulus in isolation. The thermal stimulus alone, i.e. surface at 35 °C, elicited 1.57% of mosquitoes to land on the target, the visually conspicuous target 7.98%, and host odour 19.14%, while when all three stimuli where presented simultaneously, 66.47% of the mosquitoes landed on the target. Host odour and the visual cue when presented simultaneously, elicited 41.60% of the mosquitoes to land, while 52.77% landed when they were presented with host odour and the thermal cue. Ultimately, in absence of host odour, when the visual and thermal cue were combined, only 6.8% of the mosquitoes landed on the target surface.

**Figure 4:**
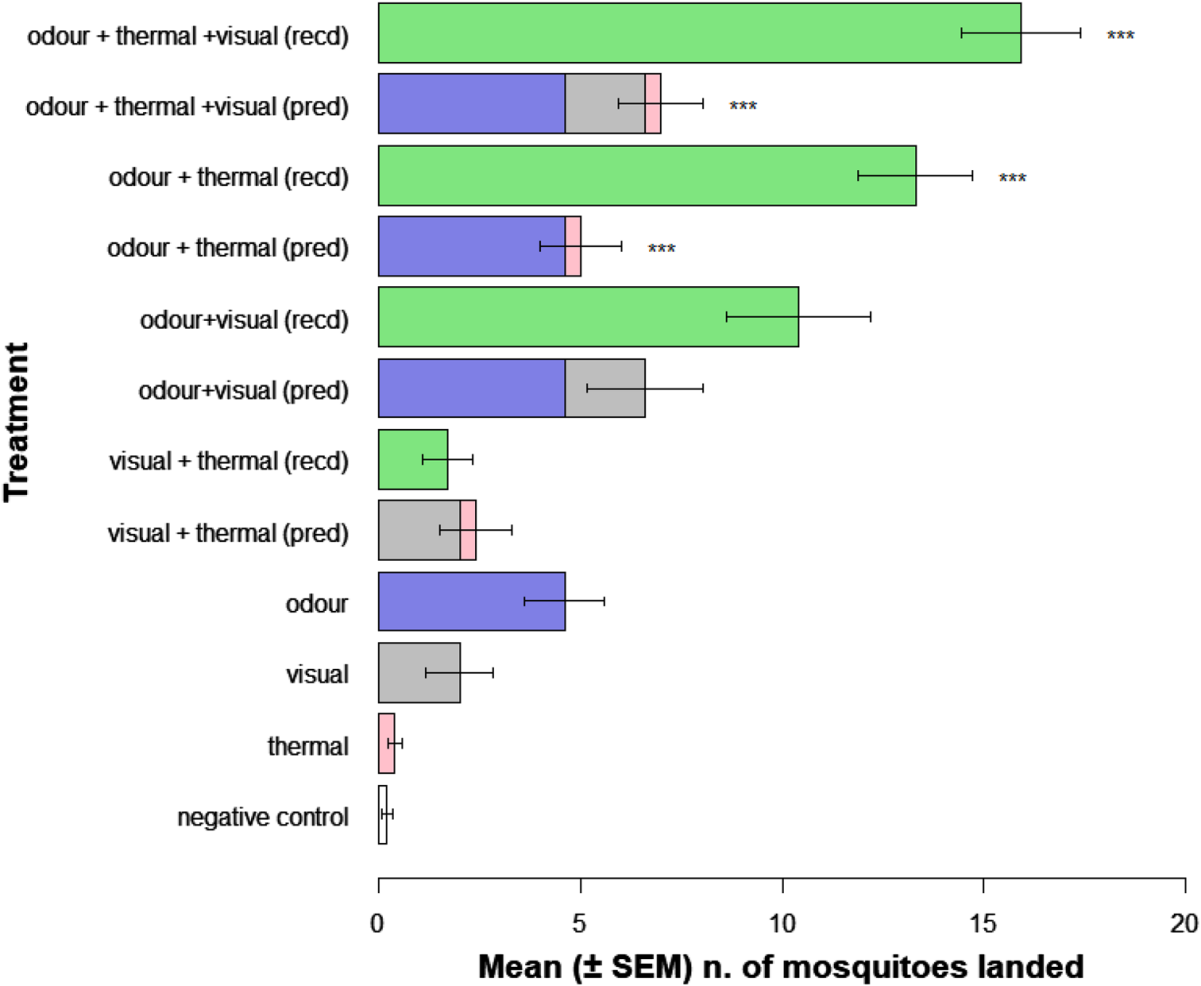
Predicted and recorded effects of host-associated stimuli on the mean landing response (± SEM) of host-seeking An. coluzzii, when stimuli were presented alone and in combination. Negative control is set based on the number of mosquitoes landing on a target when no thermal, visual or odour stimuli are presented. Recd, recorded mean from experiments; pred, additive predicted mean derived from simulated data set produced using recorded baseline values. ***Denotes significant difference at *P* < 0.001 between the recorded effect and the predicted effect for each respective treatment.

To understand the role of the different host-associated stimuli when presented in combination with other stimuli, we compared the number of mosquitoes that landed when exposed to different treatments with the number of mosquitoes expected to land if the effect of each stimulus in that combination would act in an additive manner (Fig. 4).

The most notable finding of our study is indicated in Fig. 4, where it can be seen that the recorded effect of the host-associated cues presented together exceeded considerably the effect that would have resulted by a simple sum of the singular effects of each stimulus. The recorded number of mosquitoes that landed when the three host-associated cues were presented simultaneously (Mean ± SEM = 15.9 ± 1.05) was significantly higher when compared to the number of mosquitoes that were expected to land if the effect was given by the addition of singular effects of each stimuli (Mean ± SEM = 7.0 ± 1.48) (χ^2^ = 24.15, d.f. = 1, *P* < 0.001). Similarly, the odour cue acted synergistically with the thermal cue in eliciting landing behaviour, as the recorded number (Mean ± SEM = 13.3 ± 1.41) significantly exceed the expected number given by the sum of the effect of the odour cue and the effect of the thermal cue (Mean ± SEM = 5.0 ± 1.01) (χ^2^ = 26.19, d.f. = 1, *P* < 0.001). However, we found no synergism between odour and visual cues (Mean ± SEM recorded landings = 10.4 ± 1.77, expected = 6.6 ± 1.43, χ^2^ = 2.84, d.f. = 1, *P* = 0.09) or thermal and visual cues (Mean ± SEM recorded landings recorded = 1.7 ± 0.62, expected = 2.4 ± 0.90, χ^2^ = 0.42, d.f. = 1, *P* = 0.52).

## Discussion

The findings reported here demonstrate for the first time a synergistic effect between odour, visual and thermal cues in eliciting landing behaviour in host-seeking *An. coluzzii* females. When all the host-associated cues were provided, the total number of mosquitoes landing on the surface was more than twice the number that would have been theoretically expected to land if the response was merely additive, based on the landing response attributable to each cue individually. A synergistic effect was also recorded for the odour and thermal cue, which indicates the ability of mosquitoes to integrate different types of sensory information ahead of a behavioural response. There are many advantages in using different cues that signal the same resource to produce a behavioural response. Sensitivity to a stimulus may be increased by integrating information derived from other cues, thus enhancing the response to the first cue^33,34^. Relying on several cues may also allow the insect a parsimonious use of sensory organs^33^, acquiring the most accurate information in the least costly way^35^. In environmental conditions where one cue may not be detectable, the presence of another cue, carried on a parallel sensory system, might still provide sufficient information to allow successful resource location. For instance, mutant mosquitoes that had a single sensory modality disrupted were still able to detect a host or target^25,29^.

Many host-seeking insect species rely on the integration of information which derives from different stimuli to locate their host. For example, some parasitic wasps use vibrational and visual cues when host-seeking^36^. Bark beetles flexibly integrate multimodal sensory information at a close-range (≤ 2 m) to regulate their landing behaviour^37^. Triatomines also integrate multimodal signals, where different contexts are defined by a specific combination of cues^33^. The observed behavioural integration described in our study suggests that host-seeking *An. coluzzii* adopt a similar resource-searching strategy. Recent studies suggest that for host-seeking mosquitoes, sensory integration occurs centrally, i.e. in the mosquitoes’ brain, rather than peripherally^29^. This has been corroborated for the visual-olfaction interaction using calcium tracing imaging^38^. It remains to be seen if a similar mechanism is used for the heat-olfaction interaction.

Our experiments revealed a non-synergistic effect between odour and visual cues. Visual cues are thought to evoke a range of different behavioural responses in mosquitoes. The optomotor mechanism, which was first reported in diurnal/crepuscular mosquitoes^39^ and later in nocturnal mosquitoes^40^ enables mosquitoes to spatially orient themselves relative to visual cues in the environment. However, visual cues may also be associated with potential hosts, and so can be attractive depending on the insect's physiological condition, the presence of other host-associated stimuli and the precise nature of the visual cue. In the present study the target was considerably larger when compared to the black tiles that were provided on the wind tunnel floor to facilitate optomotor anemotaxis, and additionally, the target could also present other host cues. Thus, we expect that in accordance to findings of a previour study that used a similar experimental set up^15^, the black tiles were not attractive to mosquitoes. When flying close to an object, visual cues may elicit an avoidance mechanism, i.e. flying either to the sides or upwards to avoid the object that is perceived as a barrier^14,15,41,42^. On the other hand, when additional host cues are presented, the visual avoidance mechanism is suppressed and visual cues act as attractants. This was corroborated in two laboratory studies^15,30^. It is worth noting that in these two studies, mosquitoes exposed to host odour were recorded hovering above a visually conspicuous surface without alighting on it, thus suggesting that other cues were missing. In a female mosquito located within centimetres of a visually conspicuous body, the frontal and lower field of view would be completely occupied by the view of the body. Thus, this stimulus would likely not provide any significant information that suggests that the body is in fact a host, and so would be unlikely to trigger the landing behaviour. Furthermore, nocturnal mosquitoes that feed inside human dwellings are expected to operate under very limiting light conditions, thus it is no surprise that the landing response is not governed by visual cues alone. We propose that in the context of host-seeking, visual cues serve as attractants and their role is important in the medium to close-range phase of location, while its effect in triggering landing behaviour is likely to be negligible, due to diminished resolution at very close range. A study conducted on *Ae. aegypti* also suggested that visibility is a characteristic that increments the facility by which the target is found by odour-induced searching mosquitoes, however, the “permission to land” is elicited by other stimuli, such as warm, wet convection currents^43^. Interestingly, a recent study reported how mosquitoes might use self-induced airflow patterns to avoid collision with surfaces^44^, suggesting that other cues (such as airflow) might provide directional information during the landing phase. Further studies to explore this are recommended.

We found that the addition of the odour cue to a visually conspicuous target at host temperature produced a tenfold increase in landing if compared to the treatment without the odour. Previous research showed that *An. gambiae* females landed up to 25 times more on a surface heated to 34 °C when carbon dioxide pulses were introduced in the arena, while in the absence of the odour stimulus the warm surface was ignored^26,45,46^, suggesting that odour may gate the landing response elicited by thermal cues^29,47^. Furthermore, visual attraction in host-seeking *Ae. aegypti* proved to also be odour-gated^30^, wherein carbon dioxide modulates the response of the lobula neuropil, a region dense of synaptic connections in the mosquito’s optic lobe, to discrete visual stimuli^38^. Our results support the view by which host odour is crucial in facilitating host-seeking and landing behaviour, as mosquitoes were more prone to land on a visually conspicuous and warm object when exposed to host odour. However, even in the absence of the odour cue, mosquitoes were recovered from the landing target, indicating that despite the absence of an olfactory cue, a small proportion of the tested mosquitoes responded to only thermal and visual cues. This is supported by another study with *Ae. aegypti*, which proposed that different cues might be able to interact thereby increasing a behavioural response, while remaining independent from each other^30^. Under this view, *An. coluzzii* mosquitoes might still approach and land on a warm object, even in the absence of host odour. Nonetheless, as thermal cues are hypothesised to only be detectable within a close range from the host^10,25,26,29^, and as heat alone might not effectively signal to mosquitoes the presence of a host, where an inanimate object heated by the sun may reach host-like temperatures, it might be more advantageous for mosquitoes to only orient towards and land on warm objects that also present other host-associated cues. It has to be taken into account that objects with different visual characteristics (i.e. transparent versus black) might also have different thermal transfer properties. To limit this issue in this study, the black panels that provided the visual cue where positioned underneath the target where they were not heated, as the target only emitted heat from its upper surface, thus convection currents produced by the upper heated surface of the target were unlikely to be altered.

Although fewer mosquitoes were recovered from the target surface at 45 °C, no difference was found with the number recovered on the surface at 35 °C, suggesting that mosquitoes did not differentiate between temperatures. This disagrees with a previous study on *An. gambiae*, where mosquitoes significantly preferred landing on targets at host temperature (34 ± 2 °C) compared with targets at lower (27 °C) and higher (41 °C) temperatures^48^. Two other studies which were conducted in still air using *Ae. aegypti* recorded a heat avoidance behaviour for high temperatures (40 °C or above), while both studies recorded a preference (intended as either directional choice or time spent closer to the thermal cue) for temperatures closer to human body temperature^9,24^. As the study presented here was conducted in a wind tunnel with flowing air, it is possible that the moving air might have contributed to alter the thermal cue given by the heated target, not by altering the surface’s temperature as this was maintained stable throughout the assay, but by cooling the temperature of the air surrounding the target. This might in part explain the differences between our results and previous studies. On the other hand, our results are in accordance with behaviour recorded on other blood-sucking insects (e.g. triatomines), that exhibit host-seeking behaviours in response to objects at temperatures from 2 °C above ambient temperature and up to 47°C^33^. Further studies, which should be carried out taking into account air movement, are needed to define the upper and lower limit of temperatures that elicit a landing behaviour in this mosquito species.

In summary, the strong synergistic effect between odour, visual and thermal cues indicate the robust interaction of these factors, which incremented the landing response. This has important implications for practical vector control interventions. Understanding the role of different cues used in triggering attraction and landing may be essential for the development or improvement of tools for delivering insecticides on contact, as well as traps for surveillance and control^49^. By unravelling tsetse response to different cues and their specific characteristics, researchers were able to identy key components that were then incorporated into an improved vector control approach^6^. The results described here bring new information that could be used to modify surveilance and control tools against mosquitoes, particularly where landing or contact is required. As demonstrated in this study, the addition of a visual, but more importantly, of a thermal cue in an odour releasing trap could greatly increase the number of mosquitoes caught, improving the efficiency of control methods^42,50,51^.

Taken together, the results reported here support the view that considers landing behaviour as a result of a complex series of stimuli integration, where the information deriving from different stimuli is integrated to permit a flexible, yet highly accurate and context-relevant behaviour.

## Methods

### Mosquitoes

A colony of *An. coluzzii* was established at NRI’s laboratory in 2017 using eggs derived from a colony at Institut de Recherche en Sciences de la Santé, Burkina Faso, which originated from wild gravid females collected in Vallée du Kou, Burkina Faso, 11°23′14"N, 4°24′42"W. Mosquitoes were identified to species level by PCR. Rearing of the colonies was carried out following established protocols^15^.

### Wind tunnel

The experiment was carried out in a wind tunnel (1.2 m tall × 1.2 m wide × 2 m long)^15^ (Fig. 1) which allowed mosquitoes to execute flight manoeuvres and reduced the constraining effects caused by the walls of small flight arenas used in other studies^26,52^. The arena was kept at 25 ± 2 °C and 65 ± 5% RH, and the air was drawn at the upwind end by an impelling fan from outside the building, to avoid using room air which accumulates human emanations^53^. Before entering the flight arena, the air was purified by passage through activated charcoal filters, heated, humidified and pushed through a screen of brushed cotton to create a laminar airflow. The air was pulled out of the laboratory room by an extractor fan at the downwind end. Thus a constant airflow of approximately 0.2 m s^−1^ was maintained in the flight arena^15,26,28,54^. During the experiments, the only source of illumination consisted of a series of warm white LEDs which were placed in an even array on the laboratory floor, below the floor of the flight arena, and provided a homogenous light level of 0.001 W m^−2^, similar to full moonlight illumination^55^. The wind tunnel wall and floor panels consisted of opal Perspex, which diffused the incoming light. To permit mosquitoes to orient themselves and navigate using the optomotor mechanism, nine small visually conspicuous squares (10 cm per side) and two large squares (20 cm per side) were placed randomly on the floor of the wind tunnel. Mosquitoes were released at the downwind end of the wind tunnel, from a release cage (15 × 15 × 15 cm) positioned at the centre of the crosswind axis (60 cm from the walls) and approximately 35 cm above the wind tunnel floor, and therefore centred with the odour stimulus releasing point.

### Landing target

To test landing responses which could incorporate different combinations of thermal and visual stimuli, a landing target was designed based on a commercially available transparent heated glass unit (E-GLAS sample—Saint Gobain, UK). The unit (3 × 40 x 30 cm) consisted of two sheets of glass and a layer of electrically conductive material in the middle, which allowed the glass surface to be warmed to up to 50 °C via mains power supply. During experiments, the surface temperature of the landing target was controlled using a thermostat (ReptiZoo, China) which maintained the desired temperature (± 1 °C). To achieve a highly accurate temperature control, a layer of heat sink paste (RS Components, UK) was interposed between the thermostat probe and the glass surface. At the start and end of each assay, the temperature of the surface was thoroughly scanned with an infra-red laser gun thermometer to ensure that the temperature was within ± 2 °C from the desired experimental temperature. Visual properties of the target could also be controlled; the glass could be either left transparent to provide a very low contrast stimulus or made highly visually conspicuous with the addition of opaque black plastic secured underneath the glass.

To capture mosquitoes landing on the target, the surface was covered by a layer of thin (< 1 mm) adhesive film (FICSFIL Barrettine, UK), which consists of a transparent plastic sheet coated with a transparent layer of strong glue. The landing target was positioned horizontally (flat) on the floor of the wind tunnel, at the centre of the crosswind axis (40 cm from the lateral walls) and 30 cm from the upwind end of the flight arena.

### Odour treatment

Human host odour, comprising a combination of human skin odour and carbon dioxide, was used as the olfactory stimuli. Human foot odour was sourced from 15 denier sheer knee-high 100% polyamide-nylon socks worn for 24 h by the experimenter (MC), who voluntarily consented to donate the worn socks. No ethical approval was required for this study as no data was collected about the experimenter. To limit changes in body odour, the socks were worn by the same volunteer^56^. One hour prior to wearing the socks, the volunteer washed her feet with water and fragrance-free soap. Throughout the duration of the experiment, the volunteer abstained from consuming food with spices and alcohol, did not use perfumes, perfumed soaps and clothes detergents, as these substances can affect the collected odour^57–59^. The worn socks were used in experiments for a maximum of one week, when not in use were kept in a sealed zip-lock bag at -20 °C to minimize variation of the odour components^54,60^. In addition to human body odour, carbon dioxide at approximately the concentration in human breath^56^ (flow rate of 5 L min^−1^, approx. 4.5% concentration) was also offered. The tube releasing carbon dioxide was positioned in the centre of the crosswind axis, 3 cm behind the netting and at 25 cm high above the wind tunnel floor, while the socks were presented in the same position but at 40 cm above the arena floor. Both the carbon dioxide releasing method and the wind tunnel conditions used in this study were comparable with conditions used in a previous study^15^. Translating this information into our study, it follows that the landing target was positioned in alignment with and directly below this plume, with the bottom edge of the plume modelled to touch the landing target.

### Experimental procedure

A new group of non-blood fed, four to twelve days old female mosquitoes was used for each bioassay. Four to five hours before the commencement of assays, sugar feeders were removed from adult cages. The mosquitoes were collected between one and two hours before assays using a mouth-aspirator and were kept in small WHO tubes (4.5 cm diameter and 12 cm height) in darkness to allow the eye to adapt to low light levels^61,62^. The mosquitoes were transferred to the release cage and were given five minutes to habituate to the wind tunnel environment, after which the cage was gently opened to avoid disturbing the mosquitoes and operated remotely to avoid introducing human odours into the arena. In assays requiring it, the odour treatment started immediately after the release cage was opened. Thirty minutes later the bioassay was terminated, the release cage door was closed, and the number of mosquitoes recovered in different parts of the wind tunnel was counted as follows: number in the release cage, number in the wind tunnel beside the release cage and landing target, and number on the landing target. The target surface was divided into four equal quadrants and the number of mosquitoes caught on each quadrant was recorded for each replicate. The number of mosquitoes considered activated was designated as the number found in the wind tunnel plus the number found on the landing target.

Twelve different treatments were tested (Table 2), representing all possible combinations of the following factors: visual variables (either transparent or visually conspicuous), odour variables (presence or absence of host odour) and thermal variables (the target was either at ambient temperature at 25 °C, human skin temperature at 35°C^46,63^, or a high temperature at 45 °C). The twelve treatments were tested in a randomized order, between and within days, to exclude the effect of testing sequence.

**Table 2:** Summary of the twelve tested treatments which resulted from all possible combinations of the three studied host-associated cues.

Treatment	Visual cue	Thermal cue	Odour cue
1 (negative control)	Transparent	25 °C	Absent
2	Transparent	35 °C	Absent
3	Transparent	45 °C	Absent
4	Black	25 °C	Absent
5	Black	35 °C	Absent
6	Black	45 °C	Absent
7	Transparent	25 °C	Present
8	Transparent	35 °C	Present
9	Transparent	45 °C	Present
10	Black	25 °C	Present
11 (positive control)	Black	35 °C	Present
12	Black	45 °C	Present

Prior to the commencement of sets of replicates with no odour cue, the wind tunnel surfaces were washed with deionised water, then wiped with 100% ethanol, and left to dry. All fabric components of the wind tunnel were washed at high temperature with a fragrance-free detergent (Surcare, UK). Furthermore, clean surgical gloves were worn at all times when touching the equipment, to minimize contamination of human skin odour^26^.

Secondary factors such as mosquito age, weather, and atmospheric pressure were introduced as co-variants in statistical analysis and found to not have a significant impact in either the activation rate or number landing on the target. Thus, they were excluded from further analysis.

### Statistical analysis

Statistical analysis and data visualisation were performed using R (version 3.6.0, R Development Core Team, 2013). The R packages used were “multcomp” for Tukey tests^64^, and “MASS” for negative binomial generalised linear models (GLMs)^65^.

The data were analysed using GLMs, which can accommodate non-Gaussian data, with trap visibility, trap surface temperature, and presence or absence of host odour as factors. For each replicate, data on activation was taken as the proportion of mosquitoes activated over the total amount of mosquitoes released, for which a GLM with quasibinomial errors and a logit link was used. The landing analysis was carried out on the number of mosquitoes that landed on the target surface, and for that a GLM with negative binomial errors and a log link was used. A three-way analysis of deviance was used to assess differences in numbers of mosquitoes found in different parts of the wind tunnel following different treatments. Multiple comparisons of means, using Tukey contrast to allow for corrections against inflation of Type I errors were carried out to compare results from treatments that had different combinations of factors. Landing proportion on different quadrants was analysed using a one-way analysis of deviance and then further assessed with a Chi-square test to pair-compare the number caught on different sections of the landing target.

To determine whether the effect of two or more stimuli on the landing was synergistic or additive, we needed to compare the observed results with a hypothetical data set that reflected an additive effect. To do this, the recorded numbers of landings on treatments where two or more stimuli where presented together were compared with the predicted numbers of mosquitoes that were expected to land if each cue acted in an additive manner. The expected additive landing numbers were derived from assays where each singular component was tested, with these results added to give a predicted additive effect. For example, to create a simulated data point for the additive effect of all three cues presented together, the number of mosquitoes recovered from an assay where only the visual cue was offered was added to the number of landings from an assay where only odour was provided and the number of landings from an assay where only the thermal cue was presented. To select which data points to use to create the simulated data set, the assays were grouped together according to closest chronological proximity. This limited the effects of any potential covariants that fell outside of experimental control. In this way, a simulated data set of ten points was constructed for each of the four potential treatment combinations (i.e. a simulated data set for all three cues, for the visual plus odour cues, for the visual plus thermal cues, and for the thermal plus odour cues). All data (recorded from experimental observation and predicted from the simulated data sets) were tested in a GLM with negative binomial errors and a log link. An analysis of deviance was used to assess if the recorded mean number of landed mosquitoes in treatments where cues were presented together was greater than the mean number of mosquitoes expected to land if the total effect of those stimuli was the additive sum of the effect of the individual stimuli.

## Data Availability

Data and R codes are deposited in Open Science Framework and can be accessed following this link: https://osf.io/gwpt6/?view_only=fc2cba95dda5460686e4ffe49f5a1fff.

## References

[1] WHO. Global vector control response 2017–2030 (2017).

[2] Bhatt S (2015). The effect of malaria control on *Plasmodium falciparum* in Africa between 2000 and 2015. Nature.

[3] Sokhna C, Ndiath M, Rogier C (2013). The change in mosquito vector behaviour and the emerging resistance to insecticides will challenge the decline of malaria. Clin. Microbiol. Infect..

[4] Thomsen EK (2017). Mosquito behavior change after distribution of bednets results in decreased protection against malaria exposure. J. Infect. Dis..

[5] Ranson H, Lissenden N (2016). Insecticide resistance in African *Anopheles* Mosquitoes: A worsening situation that needs urgent action to maintain malaria control. Trends Parasitol..

[6] Torr SJ, Vale GA (2015). Know your foe: Lessons from the analysis of tsetse fly behaviour. Trends Parasitol..

[7] Ferguson HM (2010). Ecology: A prerequisite for malaria elimination and eradication. PLoS Med..

[8] Zwiebel LJ, Takken W (2004). Olfactory regulation of mosquito-host interactions. Insect Biochem. Mol. Biol..

[9] Zermoglio PF, Robuchon E, Leonardi MS, Chandre F, Lazzari CR (2017). What does heat tell a mosquito? Characterization of the orientation behaviour of *Aedes aegypti* towards heat sources. J. Insect Physiol..

[10] Cardé RT (2015). Multi-cue integration: How female mosquitoes locate a human host. Curr. Biol..

[11] Gillies M (1980). The role of carbon dioxide in host-finding by mosquitoes (Diptera: Culicidae): a review. Bull. Entomol. Res..

[12] Costantini C (1998). Odor-mediated host preferences of West African mosquitoes, with particular reference to malaria vectors. Am. J. Trop. Med. Hyg..

[13] Takken W, Knols BGJ (1999). Odor-Mediated Behavior of Afrotropical Malaria Mosquitoes. Annu. Rev. Entomol..

[14] Cardé, R. T. & Gibson, G. Host finding by female mosquitoes: mechanisms of orientation to host odours and other cues. In *Olfaction in Vector-Host Interactions* 115–141 (2010). 10.3920/978-90-8686-698-4

[15] Hawkes F, Gibson G (2016). Seeing is believing: The nocturnal malarial mosquito *Anopheles coluzzii* responds to visual host-cues when odour indicates a host is nearby. Parasit. Vectors.

[16] Davis EE, Sokolove PG (1975). Temperature responses of antennal receptors of the mosquito, *Aedes aegypti*. J. Comp. Physiol..

[17] Wang G (2009). *Anopheles gambiae* TRPA1 is a heat-activated channel expressed in thermosensitive sensilla of female antennae. Eur. J. Neurosci..

[18] Maekawa E (2011). The role of proboscis of the malaria vector mosquito *Anopheles stephensi* in host-seeking behavior. Parasit. Vectors.

[19] Bohbot JD, Sparks JT, Dickens JC (2014). The maxillary palp of *Aedes aegypti*, a model of multisensory integration. Insect Biochem. Mol. Biol..

[20] Bar-Zeev M (1960). The location of hygroreceptors and moisture receptors in *Aedes aegypti* (L.). Entomol. Exp. Appl..

[21] Meijerink J, Braks MAH, Van Loon JJA (2001). Olfactory receptors on the antennae of the malaria mosquito *Anopheles gambiae* are sensitive to ammonia and other sweat-borne components. J. Insect Physiol..

[22] Khan AA, Maibach HI (1966). Quantitation of effect of several stimuli on landing and probing by *Aedes aegypti*. J. Econ. Entomol..

[23] Wright RH, Kellogg FE (1962). Response of *Aedes aegypti* to moist convection currents. Nature.

[24] Corfas RA, Vosshall LB (2015). The cation channel TRPA1 tunes mosquito thermotaxis to host temperatures. Elife.

[25] Greppi C (2020). Mosquito heat seeking is driven by ancestral cooling receptor. Science (80-).

[26] Spitzen J (2013). A 3D analysis of flight behavior of *Anopheles gambiae sensu stricto* Malaria Mosquitoes in response to human odor and heat. PLoS ONE.

[27] Gibson G, Torr SJ (1999). Visual and olfactory responses of haematophagous Diptera to host stimuli. Med. Vet. Entomol..

[28] Lacey ES, Cardé RT (2012). Location of and landing on a source of human body odour by female *Culex quinquefasciatus* in still and moving air. Physiol. Entomol..

[29] McMeniman CJ, Corfas RA, Matthews BJ, Ritchie SA, Vosshall LB (2014). Multimodal integration of carbon dioxide and other sensory cues drives mosquito attraction to humans. Cell.

[30] Van Breugel F, Riffell J, Fairhall A, Dickinson MH (2015). Mosquitoes use vision to associate odor plumes with thermal targets. Curr. Biol..

[31] Baik LS (2020). Circadian regulation of light-evoked attraction and avoidance behaviors in daytime- versus nighttime-biting mosquitoes. Curr. Biol..

[32] Cooperband MF, Allan SA (2009). Effects of different pyrethroids on landing behavior of female *Aedes aegypti*, *Anopheles quadrimaculatus*, and *Culex quinquefasciatus* mosquitoes (diptera: culicidae). J. Med. Entomol..

[33] Guerenstein PG, Lazzari CR (2009). Host-seeking: How triatomines acquire and make use of information to find blood. Acta Trop..

[34] Lehane MJ (2005). The Biology of Blood-Sucking in Insects.

[35] Fawcett TW, Johnstone RA (2003). Optimal assessment of multiple cues. Proc. R. Soc. B Biol. Sci..

[36] Fischer S, Samietz J, Wäckers F, Dorn S (2001). Interaction of vibrational and visual cues in parasitoid host location. J. Comp. Physiol. A Sens. Neural. Behav. Physiol..

[37] Campbell SA, Borden JH (2006). Close-range, in-flight integration of olfactory and visual information by a host-seeking bark beetle. Entomol. Exp. Appl..

[38] Vinauger C (2019). Visual-Olfactory integration in the human disease vector mosquito *Aedes aegypti*. Curr. Biol..

[39] Kennedy JS (1940). The Visual Responses of Flying Mosquitoes. Proc. Zool. Soc. London.

[40] Gibson G (1995). A behavioural test of the sensitivity of a nocturnal mosquito, *Anopheles gambiae*, to dim white, red and infra-red light. Physiol. Entomol..

[41] Cribellier A (2018). Flight behaviour of malaria mosquitoes around odour-baited traps: capture and escape dynamics. R. Soc. Open Sci..

[42] Cribellier A (2020). Lure, retain, and catch malaria mosquitoes. How heat and humidity improve odour-baited trap performance. Malar. J..

[43] Kellogg FE, Wright RH (1962). Studies in mosquito repellency. IV. The effect of repellent chemicals. Can. Entomol..

[44] Nakata T (2020). Aerodynamic imaging by mosquitoes inspires a surface detector for autonomous flying vehicles. Science (80-).

[45] Kröber T, Kessler S, Frei J, Bourquin M, Guerin PM (2010). An in vitro assay for testing mosquito repellents employing a warm body and carbon dioxide as a behavioral activator. J. Am. Mosq. Control Assoc..

[46] Healy TP, Copland MJW (1995). Activation of *Anopheles gambiae* mosquitoes by carbon dioxide and human breath. Med. Vet. Entomol..

[47] Webster B, Lacey ES, Cardé RT (2015). Waiting with bated breath: opportunistic orientation to human Odor in the Malaria Mosquito, *Anopheles gambiae*, is modulated by minute changes in carbon dioxide concentration. J. Chem. Ecol..

[48] Healy TP, Copland MJW, Cork A, Przyborowska A, Halket JM (2002). Landing responses of *Anopheles gambiae* elicited by oxocarboxylic acids. Med. Vet. Entomol..

[49] Kline DL, Lemire GF (1995). Field evaluation of heat as an added attractant to traps baited with carbon dioxide and octenol for *Aedes taeniorhynchus*. J. Am. Mosq. Control Assoc..

[50] Hawkes FM, Dabiré RK, Sawadogo SP, Torr SJ, Gibson G (2017). Exploiting *Anopheles* responses to thermal, odour and visual stimuli to improve surveillance and control of malaria. Sci. Rep..

[51] Homan T (2016). The effect of mass mosquito trapping on malaria transmission and disease burden (SolarMal): a stepped-wedge cluster-randomised trial. Lancet.

[52] Kennedy JS, McKelvey JJ, Shorey HH (1977). Behaviourally discriminating assays of attractants and repellents. Chemical Control of Insect Behaviour Theory and Application.

[53] Clements AN (1999). The Biology of Mosquitoes Vol. 2. Sensory Reception and Behaviour.

[54] Beeuwkes J, Spitzen J, Spoor CW, van Leeuwen JL, Takken W (2008). 3-D flight behaviour of the malaria mosquito *Anopheles gambiae* s.s.s inside an odour plume. Proc. Netherlands Entomol. Soc. Meet..

[55] Young S, David CT, Gibson G (1987). Light measurement for entomology in the field and laboratory. Physiol. Entomol..

[56] Pates HV, Takken W, Stuke K, Curtis CF (2001). Differential behaviour of *Anopheles gambiae* sensu stricto (Diptera: Culicidae) to human and cow odours in the laboratory. Bull. Entomol. Res..

[57] Verhulst NO (2011). Composition of human skin microbiota affects attractiveness to malaria mosquitoes. PLoS ONE.

[58] Lefèvre T (2010). Beer consumption increases human attractiveness to malaria mosquitoes. PLoS ONE.

[59] Shirai O (2002). Alcohol ingestion stimulates mosquito attraction. J. Am. Mosq. Control Assoc..

[60] Jawara M (2009). Optimizing odor-baited trap methods for collecting mosquitoes during the malaria season in the gambia. PLoS ONE.

[61] Sato S (1957). On the dimensional characters of the compound eye of *Culex pipiens* var. *pallens* Coquillet. Sci. Rep. Tohoku Univ..

[62] Moon YM, Metoxen AJ, Leming MT, Whaley MA, O’Tousa JE (2014). Rhodopsin management during the light-dark cycle of *Anopheles gambiae* mosquitoes. J. Insect Physiol..

[63] Menger DJ, Van Loon JJA, Takken W (2014). Assessing the efficacy of candidate mosquito repellents against the background of an attractive source that mimics a human host. Med. Vet. Entomol..

[64] Hothorn T, Bretz F, Westfall P (2008). Simultaneous inference in general parametric models. Biometrical J. J. Math. Methods Biosci..

[65] Venables WN, Ripley BD (2002). Modern Applied Statistics with S.

